# Efficacy and tolerability of mirabegron in female patients with overactive bladder symptoms after surgical treatment for stress urinary incontinence

**DOI:** 10.1590/S1677-5538.IBJU.2018.0518

**Published:** 2019-09-02

**Authors:** Mete Özkidik, Alper Coşkun, Mehmet Kazim Asutay, Tuncer Bahçeci, Nurullah Hamidi

**Affiliations:** 1 Clinic of Urology, Şanliurfa Research and Training Hospital, Şanliurfa, Turkey;; 2 Department of Urology, Ankara Atatürk Training and Research Hospital, Ankara, Turkey

**Keywords:** mirabegron [Supplementary Concept], Urinary Bladder, Overactive, Solifenacin Succinate, Urinary Incontinence, Stress

## Abstract

**Purpose:**

To evaluate the efficacy and tolerability of mirabegron in females with overactive bladder (OAB) symptoms after surgical treatment for stress urinary incontinence (SUI).

**Materials and Methods:**

The study was conducted with a prospective, randomized and double-blinded design. 62 patients over the age of 40 who met the inclusion-exclusion criterias of the study were enrolled and randomly divided into two groups as Group A (mirabegron 50mg) and B (solifenacin 5mg). Patients were compared based on efficacy of treatment [Patient Perception of Bladder Condition (PPBC) scale and micturition diaries], safety of treatment (heart rate, systolic and diastolic blood pressure, adverse events), number of micturitions per day, patient’s satisfaction status after treatment [Visual Analog Scale(VAS)] and quality of life.

**Results:**

The mean age of the population was 48.2±3.8 years and the duration of OAB symptoms was 5.9±2.9 months. Baseline values for the mean number of micturitions, volume voided in each micturition, nocturia episodes, urgency and urgency incontinence episodes were 15.3±0.34, 128±3.88mL, 3.96±1.67, 5.72±1.35 and 4.22±0.69, respectively. After treatment, values for these parameters were 11.7±0.29, 164.7±2.9mL, 2.25±0.6, 3.38±0.71, 2.31±0.49 respectively. Quality of life score, symptom bother score, VAS for treatment satisfaction score, PPBC score after treatment were 66.1±0.85, 43.7±0.77, 4.78±0.14, 4.78±0.14, respectively. There were no significant differences between two groups on any parameter. However, mirabegron showed better tolerability than solifenacin, particularly after 6 months.

**Conclusion:**

Mirabegron is safe, effective and tolerable in the long-term treatment of females with OAB symptoms after surgery for stress urinary incontinence.

## INTRODUCTION

Urinary incontinence is a bothersome symptom that has a serious impact on quality of life (1). It is classified into three types as stress urinary incontinence, urgency incontinence and mixed type incontinence due to the different pathophysiological mechanisms underlying (2).

Stress urinary incontinence (SUI) is caused by the impairment of pelvic and vaginal support to the urethra due to aging and decreasing estrogen levels in the perimenopausal period (2). If pelvic floor training which contributes to the support of the female urethra fails, surgical treatment for SUI is considered (3).

Overactive bladder is a symptom complex defined by urinary urgency, with or without urgency urinary incontinence (UUI), usually accompanied by frequency and nocturia in the absence of any obvious pathology. UUI is characterized by overactive bladder (OAB) symptoms mentioned above and additionally urinary incontinence (2). It may be seen purely, without SUI, or with SUI that is classified as mixed urinary incontinence. Patients undergoing surgical treatment for SUI may experience aggravation of OAB symptoms or develop de novo UUI after the intervention (3).

Oral anti-muscarinic agents have played the main role in the treatment of UUI for several decades (4). Although UUI frequently requires long-term treatment, inadequate response and significant adverse effects of anti-muscarinics have limited their long-term use (4), also led to frequent changes in treatment schedules.

Mirabegron is a relatively new drug, an oral ß3 adrenoreceptor agonist which facilitates urine storage by relaxing detrusor muscle (5). This different mechanism of action provides mirabegron comparable efficacy to anti-muscarinics, also less side effects than them such as dry mouth, constipation, blurred vision or cognitive impairment (4). Acceptable safety and tolerability of mirabegron leads to less discontinuation by patients in long-term treatment.

After SUI surgery, OAB symptoms constitute a major problem for patients who are in hopes of being cured. Stanford et. al. (6) reviewed the complications of suburethral sling procedures, including 20 studies with a total of 1950 patients. They found an overall incidence of de novo OAB symptoms of 15.4%, ranged from 1.7% to 42% (6). The first step in the management of OAB symptoms after SUI surgery is to rule out urinary tract infection, bladder outlet obstruction, mesh erosion, foreign body in the urethra or bladder. Then, OAB symptoms can be managed as primary OAB (treated by an antimuscarinic or a ß3 adrenoreceptor agonist).

Besides, this article aims to discuss whether efficacy and tolerability of mirabegron is adequate and acceptable in female patients with OAB symptoms after surgical treatment for SUI.

## MATERIALS AND METHODS

The study was designed as a prospective, randomized and double blinded study and conducted in accordance with ethical principles derived from The Declaration of Helsinki and Good Clinical Practice. Ethical approval was obtained from the local ethical committee. In addition, a written informed consent was taken from all patients participated.

Female patients who had a history of surgery for SUI and older than the age of 40 were prospectively enrolled to the study. Patients were randomized into two groups (Groups A and B). Block design was used for randomization. Mirabegron (oral, 50mg once a day) was given to patients (n=35) in Group A whereas solifenacin (oral, 5mg once a day) was given to patients (n=36) in Group B at the first visit. Follow-up visits were performed in every 3 months till 12^th^ month.

Primary endpoints of our study were evaluation of the efficacy and safety. Efficacy and safety were both evaluated by a single urologist in each outpatient visit. In addition, number of micturitions per 24h was accepted as a primary endpoint. All data were collected at the last visit (12^th^ month). Patients who lost to follow-up were excluded from the study.

Patient Perception of Bladder Condition (PPBC) scale and micturition diaries were used for the evaluation of efficacy. PPBC is a six-point scale on which patients are asked to rate their perceived bladder condition, ranging from 1 “no problems at all” to 6 “many severe problems”. Number of micturitions, voided volume in each micturition, nocturia episodes, urgency episodes and urgency incontinence episodes were recorded in 3-day micturition diary. In addition, a Visual Analog Scale (VAS) ranging from 0 to 10 was used for the evaluation of patient’s satisfaction after treatment. Moreover, Health Related Quality of Life (HRQoL) and symptom bother score were performed to assess the improvement in quality of life.

Safety was assessed by evaluating vital signs as fever, heart rate (n/min) and blood pressure; by performing electrocardiogram (ECG) and laboratory tests (urinalysis, complete blood count, electrolytes, renal and hepatic function tests). During follow-up, all adverse events were recorded. Expected side effects of mirabegron or antimuscarinics such as cardiac arrythmias, hypertension, nasopharyngitis, dry mouth, constipation were reported in details in each visit. Indication for the treatment cessation is accepted as any life-threating event or significant decrease in patient satisfaction assessed by Health Related Quality of Life (HRQoL) questionnaire. A hypertensive event was considered as any measurement of systolic blood pressure ≥140mm Hg or any measurement of diastolic blood pressure ≥90mm Hg, additionally an increase of ≥20mm Hg in systolic blood pressure or an increase of ≥10mm Hg in diastolic blood pressure.

Prior use of antimuscarinics or any other treatments for urinary incontinence were recorded in the first visit. Because, it was mandatory to stop using all anti-incontinence treatments before the beginning of the study, those who used antimuscarinics at the last 3 months were excluded from the study. Additionally, patients who had any perioperative complication (permanent urinary retention, mesh erosion, bladder perforation or adjacent organ injury) during SUI surgery were excluded. Other drugs used for different co-morbidities such as diabetes mellitus, hypertension or any other chronic disease were permitted to be used during the study, but recorded carefully for close follow-up in case of any adverse events.

### Statistical analysis

For analysis, SPSS 22.0 (IBM Company, Chicago) was used (7). Changes in all parameters of efficacy and safety from the first visit to the final visit for both groups were analyzed by using analysis of covariance model (ANCOVA) to obtain adjusted means. For statistical significance, p-value of <0.05 was accepted.

## RESULTS

Table-1 provides an overview of the baseline characteristics of the study population. A total of 71 patients participated in the study; however. 9 were excluded due to lacking any of the visits (5 in Group-A, 4 in Group-B). The data of remaining 62 patients were evaluated. The mean age of the population was 48.2±3.8 years and similar in both groups (Table-1). Vast majority of the participants were treatment-naive patients, only 33% had a history of prior use of any drug for OAB symptoms. Patients with MUI constituted majority of the participants in the study, as only 9 patients reported de novo OAB symptoms. Mean duration of OAB symptoms was 5.9±2.9 months. The mean duration time between SUI surgery and enrollment in the study protocol was 26 months. Trans-obturator tape (TOT) and Tension-free vaginal tape (TVT) procedures were performed for SUI surgery in 48 and 14 patients, respectively. Three patients in TOT and 1 patient in TVT group described insignificant residual SUI that required lower than 2 pads daily.

**Table 1:** Characteristics and baseline values of study population.

	All patients	Group A	Group B	p value
Mean Age (years)	48.2±3.81	47.3±4.82	49.1±2.73	0.21
Patients Participated (n)	71	35	36	-
Patients Excluded (n)	9	5	4	-
Patients Evaluated (n)	62	30(48%)	32(52%)	-
Prior Use of Any Drug for OAB symptoms (n)	21(33%)	12(40%)	9(28%)	0.17
De Novo OAB symptoms (n)	9(14%)	4(13%)	5(15%)	0.28
Preexisting and Persisting OAB symptoms(n)	53(85%)	26(86%)	27(84%)	0.26
Duration of OAB symptoms(months)	5.9±2.9	6.2±2.3	5.7±3.3	0.32
Number of micturitions per 24 h, Mean±SD	15.35±0.34	15.43±0.29	15.27±0.38	0.35
Volume voided in each micturition during 3-day micturition diary(mL), Mean±SD	128.69±3.88	129.73±3.85	127.65±3.92	0.18
Nocturia episodes during 3-day micturition diary, Mean±SD	3.96±1.67	3.89±1.83	4.03±1.52	0.42
Urgency episodes during 3-day micturition diary, Mean±SD	5.72±1.35	6.24±0.73	5.21±1.91	0.24
Urgency incontinence episodes during 3-day micturition diary, Mean±SD	4.22±0.69	4.17±0.77	4.26±0.61	0.43

**OAB** = Overactive Bladder

Efficacy assessments are summarized in Table-2. Several parameters were assessed to compare the efficacy of both drugs for the treatment of OAB symptoms in women after surgery for SUI. There was no statistically significant difference between the baseline values of two groups in any parameter. In post-treatment results, Group-A showed similar results with Group-B in all parameters (Table-2). Mirabegron group provided even slightly better results for number of micturitions per 24h though the difference was not statistically significant (p: 0.37).

**Table 2:** Parameters for Efficacy Assessment.

	All patients	Group A	Group B	p value
Number of micturitions during 3-day micturition diary, Mean±SD	11.73±0.29	11.62±0.27	11.81±0.32	0.37
Volume voided in each micturition during 3-day micturition diary(mL), Mean±SD	164.71±2.96	165.73±2.94	163.69±2.98	0.19
Nocturia episodes during 3-day micturition diary, Mean±SD	2.25±0.61	2.20±0.57	2.30±0.64	0.41
Urgency episodes during 3-day micturition diary, Mean±SD	3.38±0.71	3.15±0.73	3.62±0.68	0.29
Urgency incontinence episodes during 3-day micturition diary, Mean±SD	2.31±0.49	2.27±0.47	2.36±0.51	0.42
HRQoL Score, Mean±SD	66.1±0.85	65.8±0.82	66.2±0.87	0.30
Symptom Bother Score, Mean±SD	43.7±0.77	44.1±0.79	43.4±0.75	0.22
VAS for treatment satisfaction, Mean±SD	4.78±0.14	4.72±0.16	4.84±0.12	0.39
PPBC Scale, Mean±SD	3.93±0.17	3.91±0.20	3.95±0.15	0.73

**HRQoL =** Health Related Quality of Life; **VAS** = Visual Analog Scale; **PPBC** = Patient Perception of Bladder Condition

Patient reported outcomes (PPBC and TS-VAS) all revealed that the efficacy of mirabegron was comparable to that of solifenacin. In addition, quality of life which was assessed by the responder analyses, was similar and better than the beginning in both groups.

Safety assessments are provided in Table-3 in details. Possible side effects, adverse events or any factor leading discontinuation of treatment were reported. Hypertensive events were more frequent in Group-B. A total of 12 events in 8 different patients were recorded (Table-3). All of the hypertensive events were temporary so did not require to stop the treatment. Sinus tachycardia was observed in 2 patients of Group-A. It was asymptomatic, only recorded in ECG and did not persist in the follow-up visits. QT interval prolongation was not recognized in any patients participated.

**Table 3:** Parameters for Safety Assessment.

	All patient	Group A	Group B
Hypersensitivity (n)	-	-	-
Hypertensive Event (n)	12	8	4
Cardiac Arrhythmia (n)	2	-	2
Prolonged QT Interval (n)	-	-	-
Nasopharyngitis (n)	1	1	-
Headache (n)	3	3	-
Sinusitis (n)	1	1	-
Backpain (n)	3	2	1
Arthralgia (n)	2	-	2
Diarrhea (n)	1	-	1
Constipation (n)	2	-	2
Dry Mouth (n)	11	-	11
UTI (n)	8	3	5
AUR (n)	1	-	1
Syncope (n)	1	-	1
Nephrotoxicity (n)	3	2	1
Hepatotoxicity (n)	2	-	2
Cognitive Impairment (n)	-	-	-
Side Effects in Total (n)	53	20	33

**UTI** = Urinary Tract Infection; **AUR** = Acute Urinary Retention

Nephrotoxicity and hepatotoxicity was assessed by monitoring serum creatinine levels and liver enzymes in each visit performed. Insignificant and temporary changes as slightly elevated serum alanine transaminase levels in 2 patients and creatinine levels in 3 patients, were observed but did not require to stop the treatment. No cognitive impairment was recognized in any participants of the study. Acute urinary retention (AUR) was observed in only one patient of Group-B but did not persist despite continuation of the treatment. Urinary tract infection (UTI) rates were similar in both groups. UTIs were not severe that oral antibiotics therapy was adequate in all cases.

A total of 17 patients stopped the treatment before the end of the study. In all cases, certain side effects were responsible for discontinuation rather than inadequate response. Mean duration of treatment was 10.8 months in total but significantly longer in the mirabegron group. (11.4 vs. 10.3, p:0.042) Vast majority of patients quitted participated in the solifenacin group (13 of 17, 76%) and dry mouth was the primary factor leading to the discontinuation of treatment (11 of 13 patients in Group B). Four patients discontinued mirabegron due to the side effects, particularly headache and nasopharyngitis. But in general mirabegron provided higher tolerability than solifenacin.

## DISCUSSION

Urinary incontinence has always been under debate in Urology (8). Several choices of treatment, indispensable side effects in each option and challenging clinical cases make urinary incontinence a certain point of interest for most urologists (8).

One of the most troublesome aspects of treatments for urinary incontinence is low patient satisfaction rates (9). Probably there are so many reasons for unsatisfaction of the patients but serious side effects might be the leading problem for patients to overcome in the long-term treatment of urinary incontinence.

This study aims to determine whether mirabegron is safe and effective in females who has a history of surgical intervention for SUI by comparing with solifenacin which is a widely accepted anti-muscarinic in the treatment of OAB symptoms. There are several studies in the literature that shows the efficacy of mirabegron but most of them focus on pure UUI or mixed urinary incontinence with a predominantly component of OAB symptoms. Our study focuses on a different population. After a surgical intervention for SUI, either de novo or persisting and aggravating OAB symptoms lower the satisfaction rate of patients if it is not managed properly. This clinical condition would be considered as a different situation from primary OAB due to its different etiology.

Urodynamic studies show that detrusor overactivity occur in these patients (10). The reason why detrusor activity is observed in these patients is yet to be revealed but a possible mechanism is the adaptation of detrusor muscle to the new circumstances occurred in the lower urinary tract. In other words, it may be regarded as a response by detrusor to the increased urethral resistance provided by SUI surgery. Frequently patients suffering from OAB symptoms apply to their physician after a few weeks following surgery. Probably this period of time is required for gaining detrusor overactivity particularly in de novo OAB patients.

Long-term treatment would be required in these patients to overcome OAB symptoms. Therefore, mirabegron seems to be a good option in these cases with its well tolerability.

Several anti-muscarinics have been used in the treatment of de novo or preexisting and persisting OAB symptoms after surgery for SUI since their efficacy was proven for the treatment of UUI. Among other anti-muscarinics, solifenacin would be a sensible option with its once a day usage to compare with mirabegron, in this way we tried to avoid a possible bias due to the multiple usage of another anti-muscarinic, because of its short half-life time. But the main reason of choosing solifenacin 5mg for comparison with mirabegron is its widely availability in Turkey. Some other kinds of antimuscarinics are not licensed or easily achievable in Turkey. 5mg dosage of solifenacin would be more tolerable than 10mg for the long-term treatment of OAB symptoms.

The reason why we included women over the age of 40 in our study is to exclude women who were still in desire to have a child. Both drugs in our study were category C in pregnancy, therefore we aimed to select women without an expectation of a child anymore.

Chapple et al. (4) discussed the efficacy and safety of mirabegron by comparing 50mg and 100mg dosage with tolterodine 4mg over 2444 patients in 12 month follow-up and concluded that mirabegron was safe and effective in the long-term treatment of OAB. Marcelissen et al. (3) conducted a literature review including 10 studies and 3132 patients. The study concluded that patients with MUI were more likely to develop OAB symptoms after surgery for SUI as mentioned in our study (only 9 patients developed de novo OAB symptoms).

Similarly to our study, in a retrospective study including 342 women suffering from OAB symptoms, Schiavi et al. (11) compared efficacy and tolerability of mirabegron 50mg/day with solifenacin 5mg/day. Both drugs provided significant improvements in OAB symptoms. Detrusor overactivity decreased similarly in both groups (from 58.3% to 13.1% in the solifenacin group, from 58% to 11% in the mirabegron group). However, solifenacin showed more side effects and mirabegron was recommended as a good option for long-term treatment of OAB. To our knowledge, the largest OAB study to date was conducted by Herschorn et al. (12) (SYNERGY Study) They evaluated the efficacy of combination therapy with mirabegron and solifenacin, comparing with monotherapy and placebo 3398 patients were divided into 6 groups as placebo, solifenacin 5mg, mirabegron 25mg, mirabegron 50mg, solifenacin 5mg+mirabegron 25mg, solifenacin 5mg+mirabegron 50mg. After 18 weeks, the results showed that both of monotherapies were superior in efficacy to placebo and combination therapies superior to monotherapies, and the differences were statistically significant. (p <0.05) There was no significant difference in efficacy between solifenacin 5mg/day, mirabegron 25 mg/day and mirabegron 50mg/day monotherapies. However, solifenacin had more adverse effects than mirabegron. Our results are compatible with these studies. Both drugs provided significant improvements in OAB symptoms in our study, however mirabegron showed better tolerability than solifenacin particularly after 6 months of treatment, because of the significant side effects of solifenacin, primarily dry mouth.

### Limitations of the study

Although according to our power analysis we have adequate sample size, our population is small. The main reason of this is the low socio-cultural status and rate of literacy of the female population in this region of our country. Therefore, it was hard to find sufficient number of patients who were enabled to understand and accept the study protocol. Additionally, we could include several types of antimuscarinics in our study so that a more decisive result about the efficacy and tolerability of mirabegron would be determined. However, according to the health insurance in our government, most of the patients are not enable to achieve most of the anti-muscarinics.

Twelve months seem to be adequate to find out the safety and tolerability of mirabegron, however, longer follow-up would reveal whether any tolerance may develop to its efficacy or a dose increase may be required.

Some patients may still have residual SUI after surgery. Severity of SUI as assessed by number of pads used a day is an important predictor of the surgery outcome. We were not enable to provide adequate information from the participants about this topic. Although majority of the participants benefited from surgery, severity of SUI before surgery may affect postinterventional urinary complaints so as OAB symptoms. Moreover, evaluation of prior treatments for OAB would significantly contribute to the quality of the study but the data is lacking.

The study results provided that mirabegron was an effective treatment option for OAB symptoms after surgery for SUI. Safety assessments showed similar results for both drugs but tolerability was significantly better for mirabegron particularly after 6 months of treatment when most patients using solifenacin reported to suffer from dry mouth or constipation. Relatively less and acceptable side effects of mirabegron make it a tolerable drug for long-term treatment of UUI (13).

## CONCLUSIONS

In this study, we highlight that mirabegron is safe, effective and tolerable in the long-term treatment of OAB symptoms in females after surgery for SUI. In order to provide more definitive recommendations, more studies including larger sample sizes, more types of drugs and longer periods of follow-up are required.
